# Cardiotoxicity and Heart Failure: Lessons from Human-Induced Pluripotent Stem Cell-Derived Cardiomyocytes and Anticancer Drugs

**DOI:** 10.3390/cells9041001

**Published:** 2020-04-17

**Authors:** Agapios Sachinidis

**Affiliations:** 1 Faculty of Medicine, Institute of Neurophysiology, University of Cologne, Robert-Koch-Str. 39, 50931 Cologne, Germany; a.sachinidis@uni-koeln.de; 2 Center for Molecular Medicine Cologne (CMMC), University of Cologne, Robert-Koch-Str. 21, 50931 Cologne, Germany

**Keywords:** induced pluripotent stem cells, cardiotoxicity, heart failure, genomics biomarkers, anthracyclines, anticancer therapy

## Abstract

Human-induced pluripotent stem cells (hiPSCs) are discussed as disease modeling for optimization and adaptation of therapy to each individual. However, the fundamental question is still under debate whether stem-cell-based disease modeling and drug discovery are applicable for recapitulating pathological processes under in vivo conditions. Drug treatment and exposure to different chemicals and environmental factors can initiate diseases due to toxicity effects in humans. It is well documented that drug-induced cardiotoxicity accelerates the development of heart failure (HF). Until now, investigations on the understanding of mechanisms involved in HF by anticancer drugs are hindered by limitations of the available cellular models which are relevant for human physiology and by the fact that the clinical manifestation of HF often occurs several years after its initiation. Recently, we identified similar genomic biomarkers as observed by HF after short treatment of hiPSCs-derived cardiomyocytes (hiPSC-CMs) with different antitumor drugs such as anthracyclines and etoposide (ETP). Moreover, we identified common cardiotoxic biological processes and signal transduction pathways which are discussed as being crucial for the survival and function of cardiomyocytes and, therefore, for the development of HF. In the present review, I discuss the applicability of the in vitro cardiotoxicity test systems as modeling for discovering preventive mechanisms/targets against cardiotoxicity and, therefore, for novel HF therapeutic concepts.

## 1. Introduction

Interspecies differences make animal in vitro studies insufficient for developing in vitro models for human diseases. Thus, several efforts were made to the development of human-relevant models (for a review, see Reference [1]). More recently, human-induced pluripotent stem cells (hiPSCs) were intensively applied as disease modeling to elucidate pathological mechanisms of multifactorial and monogenic diseases at the cellular level [2]. Sophisticated differentiation protocols were established allowing generation of any somatic cell type (e.g., cardiomyocytes, neurons, and hepatocytes) from hiPSCs, derived from individual patients or healthy individuals, with the hope of developing patient-specific therapies (also known as personalized medicine) (for a review, see References [1,2]). However, there are several challenges of hiPSC-based disease modeling, cell therapy, and drug discovery (e.g., the immaturity and tumorigenicity of the somatic cells) that need to be overcome for optimization of the stem-cell-based applications (for a review, see References [1,2]).

Heart failure (HF) is a major and growing public health problem worldwide, with high morbidity, mortality, and costs [3]. Although many efforts were made in drug treatment, HF is still a major cause of morbidity and mortality worldwide [4]. Until now, human-relevant investigations on understanding the mechanisms of the development of HF were hampered because of limitations of the availability of cellular systems and of diverse ethical aspects. Moreover, HF development is a long-term process that takes years for its first clinical manifestation. Finally, until now, systematic studies were not available, allowing deep insight into the networks of signal transduction pathways relevant for the HF. HF is progressively developed due to a syndrome of starting pathological events such as myocardial infarction, sustained hypertension, renal impairment, severe arrhythmia, viral infection, stressed environment, and metabolic dysregulation, or it can be developed due to genetic heart disease. Depending on the severity of the pathological conditions, a reduced heart ejection fraction occurs by the HF, and, depending on the magnitude of the loss of cardiomyocytes from the myocardium, the heart completely fails to pump blood [5,6]. Several experimental and epidemiological studies demonstrated that the prevalence of HF is significantly increased with increasing age and is higher in the elderly as compared with younger populations [3,6]. Inherited and non-inherited heart cardiomyopathies (HCMs) contribute to the development of HF. Cardiac dysfunctions by cardiomyopathies are balanced by several adaptation processes. Persistent cardiac architectural changes in HCM can result in thinning of the ventricular walls, dilation of the ventricular chamber, and decreases in cardiac output, thus leading to HF. Adaptation processes mainly occur via myocardial structural changes including an initial increase in heart mass. The dilated cardiomyopathy causes imbalanced cardiac functions, and severe HF is developed via myocardial inflammatory mechanisms [7] and heart fibrosis [8] as a clinical outcome. Many factors which induce heart injury contribute to severe HF which can be manifested by an enlargement of the ventricular chambers accompanied with a thinning of the ventricular walls due to a significant loss of ventricular cardiomyocytes (for a review, see Reference [9]). Therefore, cell death of cardiomyocytes—whether progressive or acute—is a hallmark characteristic of HF. All three types of cardiomyocyte cell death, autophagic cell death, apoptosis, and necrosis, are observed during the progression of the HF [10]. Interestingly, the elderly has a high prevalence of developing HF, which is accompanied by apoptotic, necrotic, and autophagic events resulting in a decrease both in number and in the function of cardiomyocytes. The remaining cardiomyocytes in the aging heart are hypertrophic if the HF is compensated [9,10,11]. An understanding of mechanisms involved in HF is urgently needed to develop long term therapeutic strategies for prevention of drug-associated HF. In addition, such in vitro models will contribute to discovering novel diagnostic and therapeutic biomarkers for HF. The limitations of the availability of large amounts of human cardiomyocytes (hCMs) can be solved by the invention hiPSCs, which allows the generation of (hiPSC-CMs) in an unlimited number [12]. Next, we discuss how in vitro cardiotoxicity models can be applied to discover HF-associated pathological processes.

## 2. Anticancer Drug-Induced Cardiotoxicity

Drug-induced cardiotoxicity is a major safety issue causing HF, which has to be considered during drug development and therapeutic applications. Among anticancer drugs, the anthracycline family members such as doxorubicin (DOX), daunorubicin, and mitoxantrone are known to induce cardiotoxicity and HF (reviewed in References [13,14,15]). DOX is a highly effective anticancer drug prescribed for the treatment of a variety of cancer types, including solid tumors and hematologic malignancies in both adults and children. Despite its beneficial therapeutic effects, the long-term clinical use of DOX is limited, due to its cumulative dose-dependent cardiotoxicity and HF [16]. In this context, accumulation of DOX and its metabolites in cardiac tissues of 35 patients who received DOX at any time ante mortem was demonstrated (doxorubicinol: median concentration 92 ng/g, range 0 to 484 ng/g; DOX median 58 ng/g, range 0–1665 ng/g) [17]. A meta-analysis demonstrated that treatment with anthracyclines increased the risk of cardiac death by 4.94-fold compared to non-anthracycline treatments [18]. The acute adverse effects of DOX occur within or after 2–3 days of administration and are manifested by cardiac arrhythmias and HF. The chronic side effects of DOX are largely dose-dependent. A patient may develop dilated cardiomyopathy which is typical for HF, shortly after DOX treatment termination, or dilated cardiomyopathy may even occur 10–15 years after the termination of chemotherapy. Therefore, both acute and chronic DOX administration can lead to cardiac dysfunction, cardiomyopathy, and ultimately to HF and death [19].

Multiple mechanisms such as free-radical formation, lipid peroxidation, and DNA damage were proposed to explain the cardiotoxicity of anthracyclines [13]. Additionally, interactions of anthracyclines with the DNA topoisomerase complex or directly with DNA via intercalation, resulting in disturbances in DNA replication and transcription, were extensively studied [9,16]. The dose-dependent cardiotoxicity of anthracyclines limits their therapeutic application by damaging cardiomyocytes via the formation of reactive oxygen species (ROS), DNA damage, mitochondrial damage, and apoptosis, which are discussed as key mechanisms for HF development [13,20]. Elevated levels of cardiac troponin I (cTnI) and cardiac troponin T (cTnT) in blood correlate well with myocardial injury and act as critical plasma biomarkers for the diagnosis of cardiac damage in clinical and preclinical studies [13,21,22]. However, high levels of these biomarkers occur only after cardiac damage and can be detected for only a few hours after myocardial infarction and cardiotoxic drug treatment. To avoid drug-induced cardiotoxicity in the future, there is an urgent need to develop sensitive and reliable methods to detect or predict early cardiotoxic events using simple in vitro petri dish test systems.

## 3. Introduction of an In Vitro Cardiotoxic Model to Recapitulate the Mechanisms Involved in the Development of HF

Because of ethical reasons, the study of cardiotoxicity mechanisms and the detection of biomarkers for cardiotoxicity applying heart tissues from cancer patients are not feasible. Therefore, several research groups focused on in vivo experimental models applying rats and mice (for a review, see References [23,24]). The chronical cardiotoxic effects of DOX were investigated after treatment of the animals with different concentrations of DOX for different periods. In summary, the findings of the animal studies suggested cardiotoxic mechanisms such as ROS-induced apoptosis, mitochondria damage, and inflammation [23,24].

Although primate and human primary cardiomyocytes represent highly relevant cell systems, their use is limited by ethical reasons and the limited availability [25]. Traditional approaches for toxicological testing involve extensive animal studies, thus making testing costly and time-consuming. Moreover, due to interspecies physiological differences, animal studies do not correctly predict the actual adverse effects of drug candidates on human organs. To develop more human-relevant in vitro toxicity test systems, human embryonic stem cells (hESCs) and hiPSCs were applied to predict adverse effects of different compounds on the genome [26,27,28] and epigenome level [29]. Above all, the pharmaceutical industry is struggling with the costly withdrawal of drugs from the market due to toxic effects, often related to cardiotoxicity [30,31]. Therefore, there is an urgent need for the development of a sensitive, robust, and clinically relevant in vitro system with hCMs for efficacy and safety assessment. Reproducible and large-scale production of highly purified hiPSC-CMs makes them an attractive source for human cardiotoxicity tests (reviewed in Reference [32]). hiPSC-CMs have high physiological relevance and show typical drug-induced changes in electrophysiological properties [32]. It is expected that hiPSC-CMs will increase the predictive ability of the adverse effects of potential drugs in humans and may replace or reduce cardiac safety assessment assays based on animal-derived primary cardiomyocytes or cardiac ion channel-overexpressing cell lines [32]. Next, a strategy for the development of an advanced efficient predictive cardiotoxicity model based on hiPSC-CMs, a physiological functional readout assay, and-omics biomarkers are discussed.

The generation of contractile functional hiPSCs-CMs is well established [33,34]. The hiPSCs-CMs with similar electrophysiological properties to primary hCMs consist of atrial-, ventricular-, and nodal-like cells. Therefore, it is very likely that the hiPSCs-CMs may be suitable for safety or toxicity testing and disease modeling [35,36]. Human CMs were positively tested for identifying drug interactions with different cardiac-specific channels which initiate actions potentials and determine classical clinical biomarkers, such as lactic acid as a metabolic molecule of lactate dehydrogenase (LDH), which is released into culturing medium due to cardiomyocyte injury by a cardiotoxicant [37,38,39,40,41]. However, patient-specific hiPSCs-CMs were also used for testing of cardiotoxic drugs for patient-specific safety evaluation [42]. Recently, it was reported that, like human *ether-a-go-go* related gene (hERG)-expressing HEK293 cells, hiPSC-CMs of a diseased patient also predicted drug-induced cardiotoxicity [43]. Nevertheless, a precondition of patient-specific screening of cardiotoxic drugs should firstly rely on a well-established test system with normal physiological cardiomyocytes.

**Figure 1:**
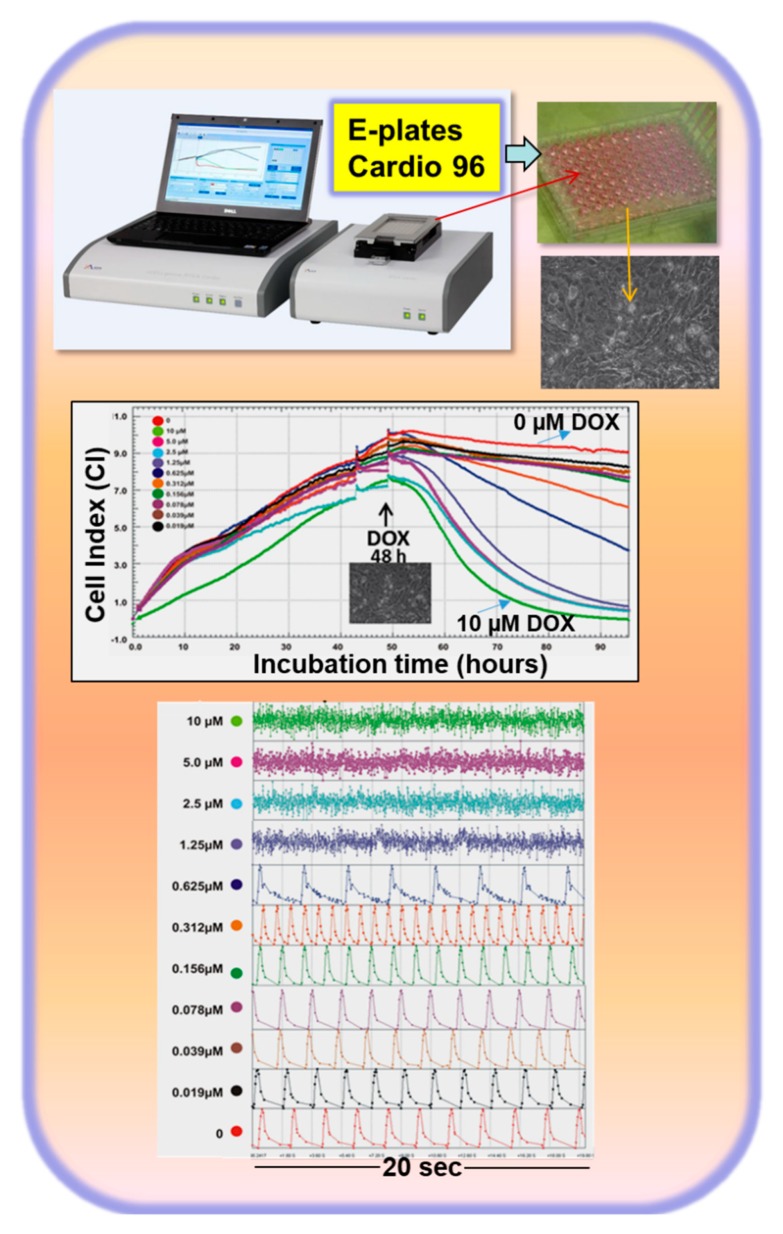
Identification of the minimal function affecting concentration (MIFAC) of doxorubicin (DOX). MIFAC is defined as a concentration affecting the function of human-induced pluripotent stem cell (hiPSC)-derived cardiomyocytes (CMs) (the beating activity) without cytotoxic effects. The so-called MIFAC concentration was determined using the xCELLigence Real-Time Cell Analyzer (RTCA) cardio system to detect DOX-induced cytotoxicity while also monitoring functional alterations of hCM by measuring the beating frequency and cell death of CMs in real time (Figure 1) [37,38,39,41].

**Figure 2:**
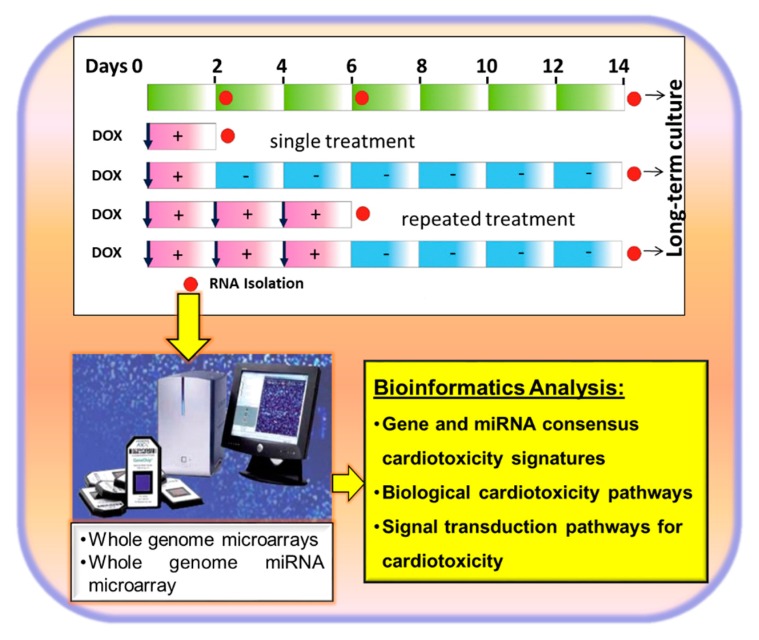
Combination of a repeated toxicity treatment protocol with transcriptomics to identify genomics biomarkers and cardiotoxicity pathways for doxorubicin.

More recently, we identified predictive genomics biomarkers of functional relevance for DOX-induced cardiotoxicity and HF using hiPSC-CMs [39]. To establish an in vitro human-relevant cardiotoxicity model, we developed an optimal strategy based on hiPSC-CMs, on anti-tumor drugs which are severe cardiotoxicants causing HF in humans, and finally on identifying -omics cardiotoxicity signatures. I believe that such a strategy will significantly accelerate the application of in vitro test systems for preclinical drug screening of cardiotoxicity and, therefore, also understanding the development of HF which is a precondition for optimal therapy. This strategy involves the following steps: (1) the identification of a concentration of a chemical that starts to affect the function of cardiomyocytes without cytotoxic effects within the 48 h of incubation; we named this concentration the minimal function affecting concentration (MIFAC) (see Figure 1); (2) classical cytotoxicity assays such as the LDH assay to confirm that MIFAC has no cytotoxicity effects; (3) -omics investigations for elucidation of detailed toxicity mechanisms using MIFAC concentrations; (4) testing of the repeated toxicity effects of a toxicant and their reversibility; (5) validation of the system with several cardiotoxic and non-cardiotoxic compounds.

The hiPSC-CMs were cultured in monolayers in E-plates Cardio 96 plates (96-multi-well plates). Changes in impedance values were converted into the cell index (CI) values which reflect the changes in cell viability and contracting activity in parallel. The software of the Real-Time Cell Analyzer (RTCA) system then allows monitoring of cytotoxicity and beating activity in parallel and in real time for several weeks. We firstly tested the well-established anti-cancer drug DOX which was extensively described to induce cardiotoxicity and HF in humans via multiple mechanisms (e.g., via ROS generation, DNA damage, and cytoskeletal disruptions). As indicated in Figure 1, after 48 h, single hCMs were attached on the multi-well plates showing a stable CI. As indicated, treatment with different concentrations of DOX for 48 h induced a time-dependent decrease of the CI index and changes in beating activity. As indicated, DOX at a concentration range of 19 to 78 nM had no effect on CI and on beating activity. Notably, the MIFAC of DOX was 156 nM since this concentration was not cytotoxic to the cells (as also confirmed by the LDH assay [39]), but significantly increased the beating activity of the hiPSC-CMs. Concentrations higher than 156 nM were cytotoxic as indicating by declining the CI and, in parallel, by not monitoring a regular beating pattern.

### 3.1. Identification of Biological Processes and Signal Transduction Pathways by Anticancer Drugs in hiPSC-Derived CMs Applying Transcriptomics

After identifying the MIFAC for DOX, we applied the protocol indicated in Figure 2.

The hiPSC-CMs were treated with a single and repeated dose of 156 nM DOX and then the medium was washed out. RNA was then isolated (two days, six days, 14 days untreated hiPSC-CMs, two days and six days treated, or two days or six days treated following washout of the drug and then isolation of total RNA). After performing the genome-wide microarray analysis with the RNA samples, more than 2000 differential expressed genes among the different conditions were identified and analyzed by comprehensive bioinformatics. In summary, the analysis demonstrated that DOX treatment preferentially decreased the expression of genes participating in cardiac contraction and pathways related to cardiomyopathies. Furthermore, DOX exposure for more than two days also deregulated genes participating in apoptosis, DNA damage, and the oxidative stress response. We identified several clusters of genes whose expression behaved completely differently, e.g., genes which were downregulated (sarcomere, myofibrils, contractile fiber, and regulation of heart contraction genes) or upregulated (stress response, p53 signaling pathway, and apoptosis genes) after two and six days of treatment with 156 nM DOX become again upregulated or downregulated toward control levels after washing out of the drug. The gene recovery ability of the hiPSC-CMs occurring after washing out of DOX was also reflected in the recovery of beating activity after washing out of DOX. These results indeed proved the capacity of the hiPSC-CMs to recover after removal of a toxicant. We further chose the most common highly deregulated genes between the two-day and six-day treatments representing key biological processes (50 downregulated genes and 34 upregulated genes), such as cardiac function or energy metabolism regulated by mitochondria, which represented the gold standard DOX transcriptome signature. Out of the 84 genes, regulation of 65 genes could also be confirmed by qPCR [39]. qRT-PCR analysis of the DOX, daunorubicin, and mitoxantrone groups displayed 35 commonly influenced genes: 27 down- and eight upregulated genes [39].

Among anticancer drugs, anthracyclines such as DOX, daunorubicin, and mitoxantrone are cardiotoxic, as manifested in the development of HF after patient treatment [13]. To identify drug class-related cardiotoxic signatures, we firstly determined the MIFAC concentrations of daunorubicin (10 nM) and 3 nM mitotraxone as described in Figure 1, and then we treated hiPSC-CMs for two days (Figure 2) and identified, 27/45 commonly downregulated genes and 8/20 commonly upregulated genes. The 27 genes are mainly involved in sarcomere architecture and the regulation of ion homeostasis. Upregulated genes were mainly associated with general stress response, and they covered stress markers such as *BAX*, *FAS*, *GPX1*, and *ZMAT3*. This observation may help to better understand cellular mechanisms underlying late apoptosis-inducing cardiac cell loss many years after anthracycline treatment. Like DOX, daunorubicin and mitotraxone belong to the anthracycline class of anti-cancer drugs. The identified 35 genes in the overlap of all three anthracyclines represent an anthracycline responsive gene consensus expression signature, which could be applied as a predictive toxicity signature for potential cardiotoxicants that act via similar mechanisms to DOX, daunorubicin, or mitoxantrone. Etoposide (ETP), an anticancer cardiotoxic drug, inhibits cell proliferation via inhibition of topoisomerase II (TopII). Recently, we performed a similar study with ETP and treated hiPSC-CMs for 48 hours with different concentrations of ETP (Figure 2). Interestingly, we also identified similar genes deregulated by ETP; downregulated genes were enriched in Gene Ontology (GO) categories like cytoskeletal organization, muscle contraction, and Ca^2+^ homeostasis, whereas most upregulated genes were enriched in GO categories like positive regulation of apoptotic processes and regulation of cell death. Growth differentiation factor 15 (*GDF15*) was also dramatically upregulated after treatment of the hiPSC-CMs with different concentrations of ETP in comparison to untreated cardiomyocytes [41]. Similarly, a strong increase in the expression level of *BAX* and *FAS* was observed.

### 3.2. Non-Coding RNAs and Cardiotoxicity

Non-coding RNAs regulate gene expression by binding to complementary regions of transcripts to inhibit their translation and/or promote messenger RNA (mRNA) degradation, thereby regulating biological processes including vertebrate development (for a review, see References [44,45,46,47]). In particular, microRNAs (miRNAs or miRs) regulate heart development and functional physiologic processes. We further extended our DOX study by analyzing miRNAs as potential cardiotoxicity biomarkers after exposing hiPSC-CMs to DOX using the same protocol as described in Figure 1 [37]. A single exposure for two days and a repeated exposure for six days to DOX resulted in deregulation of 21 miRNAs (15 upregulated and seven downregulated) and 79 miRNAs (37 upregulated and 42 downregulated), respectively. Interestingly, after washing out, only five miRNAs (out of 21) and 26 (out of 79) were identified as differentially expressed, again demonstrating the recovery potential of hiPSC-CMs after washing out of the drug. Further analysis identified 14 (10 upregulated and four downregulated) commonly deregulated miRNAs after treatment of hiPSC-CMs with 156 nM DOX for two and six days. These 14 deregulated miRNAs are predictive for early response to DOX. Among the 14 miRNAs, five miRNAs showed persistent upregulation after washing out of the drug until day 14 of incubation [37]. Among them, 10 miRNAs (hsa-miR-187-3p, hsa-miR-182-5p, hsa-miR-486-5p, hsa-miR-34a-3p, hsa-miR-486-3p, hsa-miR-212-3p, hsa-miR-4423-3p, hsa-miR-139-5p, hsa-miR-34c-3p, hsa-miR-34c-5p, hsa-miR-1303) and four miRNAs (hsa-miR-3911, hsa-miR-675-5p, hsa-miR-4298, hsa-miR-1303) were identified as overexpressed and downregulated, respectively, under the single- and repeated-dose treatments of hiPSC-CMs with DOX [37]. Among the DOX-exposed and drug washout groups, five miRNAs (hsa-miR-187-3p, hsa-miR-182-5p, hsa-miR-182-5p, hsa-miR-4423-3p, hsa-miR-34c-5p) showed persistent upregulation. We further investigated whether the 14 miRNAs deregulated by DOX could also be affected by treatment of ETP for 48 h. Treatment of hiPSC-CMs with ETP for 48 h resulted in upregulation of the expression of hsa-miR-34a-3p, hsa-miR-486-3p, and hsa-miR-4423-3p [41]. Notably, the expression level of genes and miRNAs deregulated by the anthracyclines [37,41] was not affected, even by high concentrations of cosmetic ingredients [38]. In a more general perspective, such genomics biomarkers in combination with stem-cell-based cardiotoxicity assays also become very attractive as alternative test methods for human-relevant cardiotoxicity [1] and HF.

### 3.3. Transcriptome-Based Quantification of the Cardiotoxicity Capacity of Different Compounds

To quantify the predictive capacity of our test system, the influence of the test compounds on gene expression, a “cardiotoxicity index” (CTI_84g_), was defined. CTI_84g_ expresses the ratio of the number of the deregulated genes out of the 84 which were deregulated by the gold standard cardiotoxicant DOX. The CTI_84g_ value covers functional and metabolism genes essential for an intact hiPSC-CMs function. To evaluate the effects of five cosmetic compounds, which should be non-cardiotoxic, on cell viability and hiPSC-CMs beating activity, hiPSC-CMs were cultured for 48 h with five compounds, each at three different concentrations (Figure 2), and the expression of the 84 genes deregulated by DOX was checked. As we previously described, the 84 genes participate in several processes of hiPSC-CMs such as contraction, energy metabolism, and cell death [37,38,39,41]. Our findings suggest that hiPSC-CMs exposed to 10 µM triclosan (TS), 10 µM and 3 µM triclocarban (TCC), and 200 µM 2,7-naphthalenediol (NPT) were cytotoxic and induced cardiac contractile dysfunction of hiPSC-CMs as monitored by altering the beating frequency of hiPSC-CMs dysfunction [38]. An even higher concentration of 2 µM basic red 51 (BR51) had no effects on cell viability or beating function. Interestingly, the arrhythmic beating of hiPSC-CMs during test compound incubation also recovered to basal values after a 48-h washing out period of the compounds, indicating the reversibility of the cardiotoxicity effects of a compound after removing it from the culture medium [38]. We could demonstrate that concentrations of the five cosmetic ingredients explored in this study below the MIFACs of each compound can be considered as safe to human hiPSC-CMs since the CTI_84g_ values at MIFACs were close to zero and increased with increasing concentrations of the compound [38]. The predictive capacity of the CTI_84g_ for cardiotoxicity preclinical studies was also validated using several concentrations of the ETP, another anticancer drug which was identified as a cardiotoxicant in clinical studies but at much higher concentrations, such as the gold standard DOX [41]. We validated three different concentrations of ETP below (10 µM) and above (15 and 30 µM) the MIFAC and calculated the CTI_84g_. Again, the CTI_84g_ values for concentrations below MIFACs were almost zero and increased to 0.37, 0.43, and 0.66 for 10, 15, and 30 µM, respectively. Since the CTI_84g_ value increased proportionally with the increasing concentration of ETP, we can even speculate that ETP at a MIFAC with a CTI_84g_ value of 0.37 might be less cardiotoxic than DOX, for which the CTI_84g_ is 1.0 at the MIFAC of 156 nM. Again, we confirmed that ETP concentrations at MIFAC (and higher) induced damage in the mitochondria (energy metabolism) and cardiomyocyte cytoskeleton (contraction). In conclusion, the CTI_84g_ value is a very sensitive cytotoxicity predictor [41].

## 4. Is It Possible to Identify Common Factors Playing a Role in Heart Failure and Cardiotoxicity Using In Vitro Cardiotoxicity Methods?

**Figure 3:**
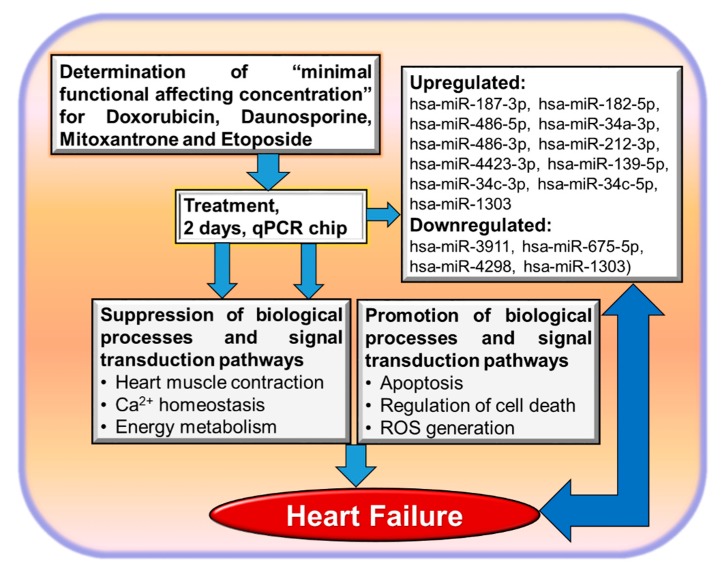
Common pathways of anticancer drug-induced cardiotoxicity, as well as microRNA biomarkers, which are relevant for the development of heart failure. Pathways were identified applying the in vitro cardiotoxicity system as shown in Figure 1 and Figure 2. As determined from in vivo animal studies [23,24], anthracyclines and ETP elicit cardiotoxic effects via reactive oxygen species (ROS)-induced apoptosis, mitochondria damage, and inflammation (upregulation of growth differentiation factor 15 (GDF15)) in hCMs [37,38,39,41].

We identified commonly deregulated genes by all the applied anticancer drugs. The identified 35 genes in the overlap of all three anthracyclines represent an anthracycline responsive gene consensus expression signature and could be applied as a predictive toxicity signature for potential cardiotoxicants that act via similar mechanisms to DOX, daunorubicin, or mitoxantrone. Downregulated genes were enriched in GO categories like cytoskeletal organization, muscle contraction, and Ca^2+^ homeostasis, whereas most upregulated genes were enriched in GO categories like positive regulation of apoptotic process and regulation of cell death. All these processes were also dysregulated by the HF (see Figure 3).

GDF15 was dramatically upregulated after treatment of the hiPSC-CMs with all the anticancer drugs applied in our test system in comparison to untreated hiPSC-CMs [39,41]. Its increased expression was accompanied by the development of HF [48,49,50] and all-cause mortality [51,52]. GDF15 was proposed as a biomarker for a diseased cell phenotype and different somatic cell injuries [53,54], as well as a prognostic biomarker for mitochondrial diseases [55]. The cardio-protective effects of GDF15 under pathological conditions were established, and GDF15 is implicated in aging and various age-related disorders such as cardiovascular diseases including HF [56]. Nevertheless, the molecular mechanisms are completely unknown. As we reported, the p53 signaling pathway and apoptosis GO terms were overrepresented in DOX- [39] and ETP-treated [41] human CMs. It is well known that two main processes are unbalanced by the HF and other cardiovascular diseases. Among the downregulated genes, several cytoskeletal structure-, channel-, and metabolism-associated genes were found as commonly regulated by all three anthracyclines [39]. In this context, we could demonstrate that the ETP-induced cardiotoxicity of hiPSC-CMs occurred via damaging the mitochondria of hiPSC-CMs, and the activation of the p53-mediated ferroptosis pathway by ETP is likely the critical pathway in ETP-induced cardiotoxicity. The apoptosis inhibitor Pifithrin-α was found to protect mouse heart injury induced by DOX [57]. We also reported that Pifithrin-α (an inhibitor of p53 transcriptional activity) [58] prevents hPSC-CMs from ETP-induced cardiotoxicity. These findings suggest that GDF15 may act as a cardio-protective factor via suppression of the DOX-induced cell death of hiPSC-CMs mediated by generation of ROS and/or suppression of the p53 signaling apoptosis pathway. In this context, it was shown that GDF15 contributes to radioresistance of cancer cells by suppressing cellular ROS [59]. Moreover, it was also suggested that GDF15 inhibits endothelial cell apoptosis in response to high glucose by inhibiting ROS overproduction [60]. Ferroptosis is a non-apoptotic cell death initiated by increased ROS production and mitochondrial deformities [61]. Liproxstatin-1 is a ferroptosis inhibitor, which was shown to protect the mouse myocardium against ischemia/reperfusion injury [62]. It was suggested that Liproxstatin-1 displays cytoprotective activity as a radical-trapping antioxidant (RTA) [63]. Recently, we also demonstrated that Liproxstatin-1 significantly accelerated the recovery of the hPSC-CMs functional properties such as beating frequency after the ETP treatment [41]. Interestingly, it was shown that treatment of the GDF15 knockout animals with lipopolysaccharide augmented the inflammatory response and aggravated septic heart and renal injury [64]. These recent findings support our hypothesis that GDF15 acts as a preventing agonist against DOX-induced heart injury.

More recently, miRNAs were recognized to play important roles in HF, and they are involved in several pathophysiological processes related to HF [45,46,47]. Moreover, specific miRNAs are also identified in blood circulation and may be used as potential diagnostic and prognostic biomarkers for HF [65]. In particular, the miR-34 family members were suggested to be involved in HF [66,67,68], and higher plasma levels of miR-34a may predict HF [69]. Consistently, the applied in vitro cardiotoxicity model also identified upregulation of the miR-34 family members under DOX treatment of hiPSC-CMs. Additionally, we identified several other miRs (Figure 3) that were discussed to be involved in heart diseases [37,41]. Notably, it was reported that the miR-34 family members were also consistently upregulated in rat and mouse heart tissues after DOX treatment [70]. Since the miR-34 family members were also upregulated in hCMs by DOX [37] and ETP [41], we suggest that the miR-34 family is one of the key regulators of the cardiotoxicity of anticancer drugs with high relevance for humans.

## 5. Conclusions and Perspectives

Induced human pluripotent stem cell (hiPSC) technologies enable the generation of human somatic cells that can be applied for prediction of toxicity in humans. Anticancer-drug treatment can initiate cardiotoxicity, and heart failure (HF) development may be considered as an episode of cardiotoxicity. The identification of pathological mechanisms in humans suffering HF is a time-consuming procedure bearing several ethical aspects hindering such studies in vivo. The application of in vitro cardiotoxicity test systems can significantly contribute to understanding the development of HF, to discovering new prognostic biomarkers, and to screening antitumor drugs for safer therapeutic applications. Several in vitro predictive genomics biomarkers of functional relevance for the HF were identified with gold standard cardiotoxicants and hiPSC-derived cardiomyocytes (Figure 3). Prospectively, the in vitro dish approach can also be applied to identify potential preventive compounds against cardiotoxicity and HF within a short period time and in a cost-effective manner. Human-relevant in vitro toxicity test systems with hiPSC-CMs in combination with genomics safe toxicity indices will significantly contribute to optimizing the anticancer patient treatment with fewer HF episodes. Moreover, anticancer drugs can be pre-screened for cardiotoxic effects of functional relevance before applications to cancer patients to avoid cardiotoxicity and, therefore, development of HF. The present in vitro approach also allows screenings of chemicals and environmental factors for potential cardiotoxicity outcomes, thus making testing less costly and time-consuming. This approach will also contribute to a significant reduction in animal studies for cardiotoxicity testing.
